# Case reports of a c.475G>T, p.E159* lamin A/C mutation with a family history of conduction disorder, dilated cardiomyopathy and sudden cardiac death

**DOI:** 10.1186/s12872-019-01282-6

**Published:** 2019-12-17

**Authors:** Tetsuro Yokokawa, Shohei Ichimura, Naoko Hijioka, Takashi Kaneshiro, Akiomi Yoshihisa, Hiroyuki Kunii, Kazuhiko Nakazato, Takafumi Ishida, Osamu Suzuki, Seiko Ohno, Takeshi Aiba, Hiroshi Ohtani, Yasuchika Takeishi

**Affiliations:** 1 Department of Cardiovascular Medicine, Fukushima Medical University, 1 Hikarigaoka, Fukushima, 960-1295 Japan; 2 Department of Pulmonary Hypertension, Fukushima Medical University, Fukushima, Japan; 3 Department of Arrhythmia and Cardiac Pacing, Fukushima Medical University, Fukushima, Japan; 4 Department of Advanced Cardiac Therapeutics, Fukushima Medical University, Fukushima, Japan; 5 Department of Diagnostic Pathology, Fukushima Medical University, Fukushima, Japan; 6 Department of Bioscience and Genetics, National Cerebral and Cardiovascular Center, Suita, Japan; 7 Department of Cardiovascular Medicine, Shiga University of Medical Science, Otsu, Japan; 8 Department of Cardiovascular Medicine, National Cerebral and Cardiovascular Center, Suita, Japan; 9 Department of Cardiovascular Medicine, Iwase General Hospital, Fukushima, Japan

**Keywords:** Lamin A/C, Dilated cardiomyopathy, Sudden cardiac death, c.475G > T, p.E159*, Case report

## Abstract

**Background:**

Patients with some mutations in the lamin A/C (*LMNA*) gene are characterized by the presence of dilated cardiomyopathy (DCM), conduction abnormalities, ventricular tachyarrhythmias (VT), and sudden cardiac death (SCD). Various clinical features have been observed among patients who have the same *LMNA* mutation. Here, we show a family with cardiac laminopathy with a c.475G > T, p.E159* *LMNA* mutation, and a family history of conduction disorder, DCM, VT, and SCD.

**Case presentation:**

A proband (female) with atrial fibrillation and bradycardia was implanted with a pacemaker in her fifties. Twenty years later, she experienced a loss of consciousness due to polymorphic VT. She had a serious family history; her mother and elder sister died suddenly in their fifties and sixties, respectively, and her nephew and son were diagnosed as having DCM. Genetic screening of the proband, her son, and nephew identified a nonsense mutation (c.475G > T, p.E159*) in the *LMNA* gene. Although the proband’s left ventricular ejection fraction remained relatively preserved, her son and nephew’s left ventricular ejection fraction were reduced, resulting in cardiac resynchronization therapy by implantation of a defibrillator.

**Conclusions:**

In this family with cardiac laminopathy with a c.475G > T, p.E159* *LMNA* mutation, DCM, SCD, and malignant VT occurred. Clinical manifestation of various atrial and ventricular arrhythmias and heart failure with reduced ejection fraction occurred in an age-dependent manner in all family members who had the nonsense mutation. It appears highly likely that the E159* *LMNA* mutation is related to various cardiac problems in the family of the current report.

## Background

Some mutations in the lamin A/C (*LMNA*) gene cause familial dilated cardiomyopathy (DCM), and the phenotype is also characterized by progressive conduction abnormalities, atrial arrhythmias, ventricular tachyarrhythmia (VT), and sudden cardiac death (SCD) [1–7]. The *LMNA* gene encodes lamins A and C, which are major components of the nuclear lamina, a dynamic protein meshwork in the nuclear membrane. The nuclear lamina has a vital role in a multitude of functions, ranging from providing structural support for the nucleus, to facilitating chromatin organization, gene regulation and DNA repair [8]. The *LMNA* mutation causes a variety of clinical illnesses, such as skeletal muscle disease, premature aging, lipodystrophies, and cardiomyopathies [9].

Approximately 6.2% of all DCM cases are caused by *LMNA* mutations [10]. DCM patients with *LMNA* mutations have poor long-term outcomes with various clinical courses, and the SCD rate is reported to be as high as 46% [11, 12]. Several reports have shown sex-specific differences in the prognostic effects of DCM patients with *LMNA* mutations [13, 14]. Three multicenter studies of patients with *LMNA* mutations in several countries suggested that male sex was an independent predictor for malignant VT [11, 13, 15]. Additionally, a study of a large cohort of *LMNA* mutation carriers demonstrated that male patients have a higher incidence of worse VT and end-stage heart failure than female patients [14]. A study by Arimura et al. found that nuclear accumulation of the androgen receptor and testosterone is associated with cardiac remodeling in DCM with *LMNA* mutations [16]. Thus, as well as the genetic identification, sex difference should also be considered for the management of therapies in individual patients with *LMNA* mutations [17].

To determine the details of the phenotype of a c.475G > T, p.E159* *LMNA* mutation (the ClinVar accession number, **SCV000996024)**, we report patients from the same family who have histories of conduction disorder, sick sinus syndrome, DCM, VT, and SCD.

## Case presentations

### Case 1

**Fig. 1:**
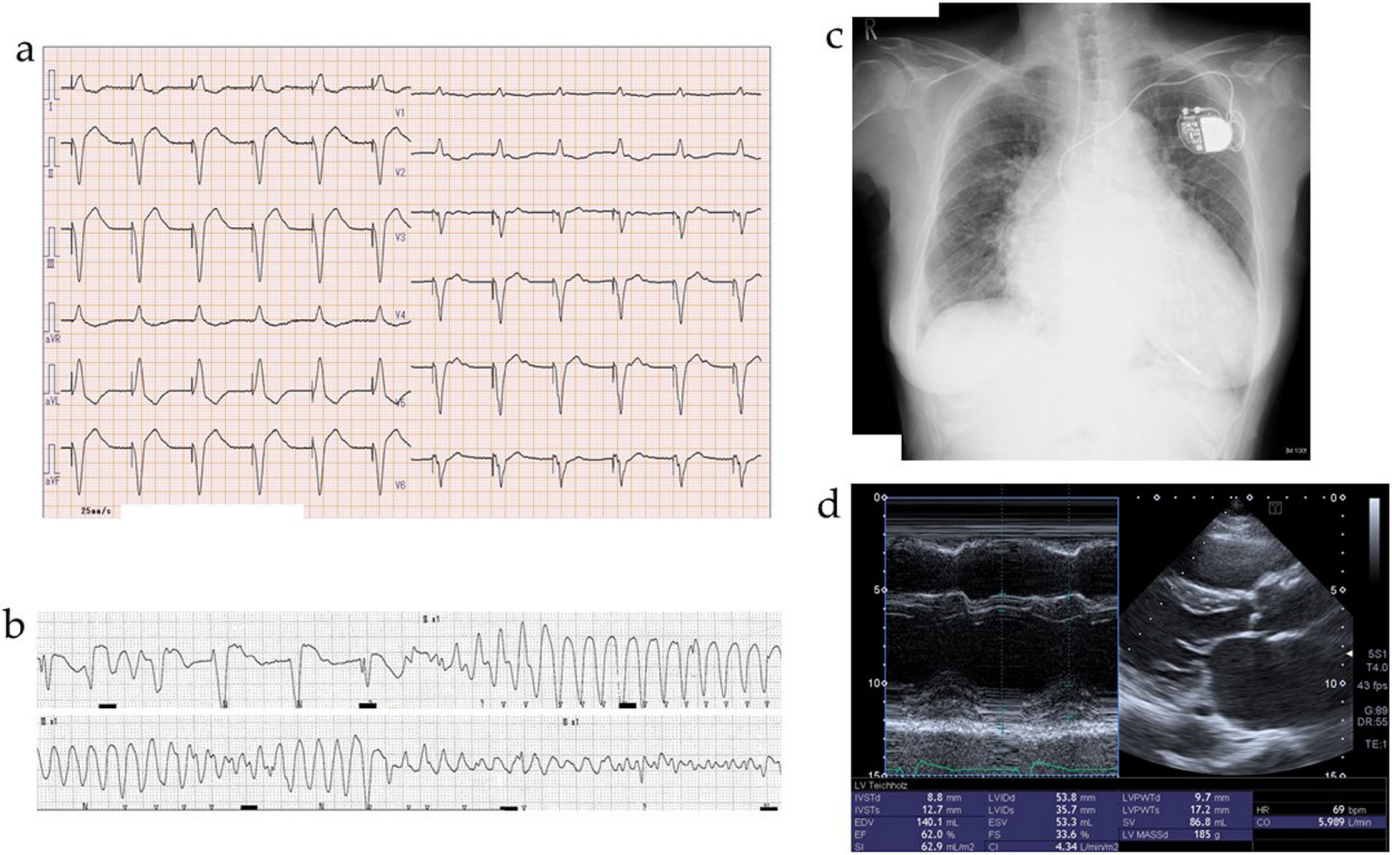
Images of Case 1 (**II-4**). **a** On admission, electrocardiogram showed pacing rhythm, a heart rate of 70 ppm, and a QTc interval of 441 msec. **b** Polymorphic ventricular tachycardia intermittently occurred. **c** Chest X-ray revealed slight congestion, with a cardiothoracic ratio of 71.0% with a pacemaker. **d** Echocardiogram showed a normal left ventricular ejection fraction of 62%, a slightly large left ventricular diastolic diameter of 53.8 mm, and a large left atrial diameter of 54 mm

A female proband (**II-4**) in her fifties suffered from atrial fibrillation with bradycardia, and was implanted with a pacemaker. About 20 years later, she was admitted to our hospital with loss of consciousness due to polymorphic VT. Consciousness was recovered via transient chest oppression. Electrocardiogram after recovery showed a pacing rhythm with a heart rate of 70 ppm and a QTc interval of 441 msec (Fig. 1a). After admission, polymorphic VT intermittently occurred (Fig. 1b), and therefore intravenous lidocaine and magnesium sulfate were administered. Furthermore, the patient was diagnosed as having influenza, and was administered laninamivir. Her laboratory findings revealed a low potassium level of 3.3 mEq/L, an increased white cell count of 11,900/μL, an increased C-reactive protein level of 2.38 mg/dL, and a B-type natriuretic peptide level of 59.0 pg/mL, as shown in Table 1. She had not been administered any medication that prolonged the QT interval. Intravenous and oral potassium was administered. Her polymorphic VT did not recur after treatment. Her chest X-ray revealed slight congestion, with a cardiothoracic ratio of 71.0% with a pacemaker (Fig. 1c). Echocardiogram showed a normal left ventricular (LV) ejection fraction of 62%, a slightly large LV diastolic diameter of 53.8 mm, and a large left atrial diameter of 54 mm (Fig. 1d).

**Table 1:** Laboratory findings on admission in Cases 1, 2, and 3

Parameters	Case 1	Case 2	Case 3
Blood count			
White cell count, /μL	11,900	6300	10,200
Red blood cell, /μL	372 × 10^4^	436 × 10^4^	414 × 10^4^
Hemoglobin, g/dL	10.9	13.9	13.7
Platelet count, /μL	21.4 × 10^4^	25.7 × 10^4^	30.1 × 10^4^
Biochemistry			
AST, IU/L	23	37	35
ALT, IU/L	14	30	38
LDH, IU/L	173	218	275
ALP, IU/L	249	180	761
Total bilirubin, mg/dL	0.8	0.7	1.2
CRP, mg/dL	2.38	0.05	1.15
BUN, mg/dL	7	14	10
Creatinine, mg/dL	0.52	0.77	0.72
eGFR, mL/min/1.73 m^2^	84.3	82.0	90.0
Sodium, mEq/L	133	139	141
Potassium, mEq/L	3.3	4.3	4.3
Chlorine, mEq/L	95	106	96
Calcium, mg/dL	8.1	–	–
IP, mg/dL	2.5	–	–
Magnesium, mg/dL	2.0	–	–
Total protein, g/dL	7.2	7.2	8.2
Albumin, g/dL	4.1	4.1	3.9
Uric acid	–	5.4	6.1
Creatine kinase, IU/L	152	152	24
CK-MB, IU/L	–	2.7	0.7
Troponin I, ng/mL	–	0.176	0.215
BNP, pg/mL	59.0	59.5	317.4
Triglyceride, mg/dL	–	74	214
HDL-C, mg/dL	–	79	40
LDL-C, mg/dL	–	75	110
FT3, pg/mL	–	2.61	2.69
FT4, ng/mL	–	2.21	1.06
TSH, U/mL	–	2.720	6.450
Glucose, mg/dL	–	98	118
HbA1c (NGSP), %	–	5.8	6.3

*AST* Aspartate transaminase, *ALP* Alkaline phosphatase, *ALT* Alanine aminotransferase, *BNP* B-type natriuretic peptide, *BUN* Blood urea nitrogen, *CK-MB* Creatine kinase MB, *CRP* C-reactive protein, *eGFR* Estimated glomerular filtration rate, *FT3* Free triiodothyronine, *FT4* Free thyroxine, *HbA1c* Hemoglobin A1c, *HDL-C* High-density lipoprotein, *IP* Inorganic phosphorus, *LDH* Lactate dehydrogenase, *LDL-C* Low-density lipoprotein, *NGSP* National Glycohemoglobin Standardization Program, *TSH* Thyroid stimulating hormone

**Fig. 2:**
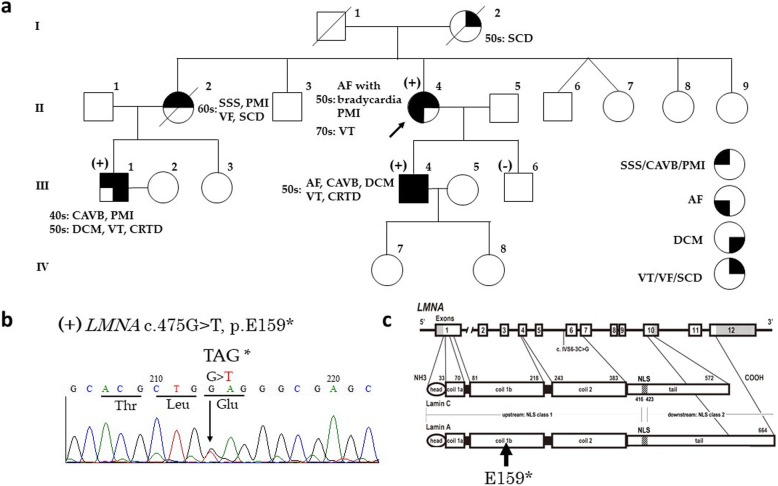
**a** Pedigree and results of Sanger sequencing. Genetic testing was performed in Case 1 (**II-4**), 2 (**III-1**), 3 (**III-4**), and the second son of the patient of Case 1 (**III-6**). An *LMNA* nonsense mutation, E159*, was identified in Cases 1, 2, and 3. The second son did not have any *LMNA* mutations. The proband is indicated by an arrow. Cases with the E159* *LMNA* mutation are shown as **(+)**, and those without the mutation are shown as **(−)**. Squares represent males, and circles represent females. **b** Direct sequencing revealed a nonsense mutation of *LMNA* (E159*) in Case 1. **c** Structure of *LMNA* (top) and lamin A (bottom) and C (middle) protein. AF, atrial fibrillation; CAVB, complete atrioventricular block; CRTD, cardiac resynchronization therapy defibrillator; DCM, dilated cardiomyopathy; *LMNA*, lamin A/C; NLS, nuclear localization signal; PMI, pacemaker implantation; SCD, sudden cardiac death; SSS, sick sinus syndrome; VF, ventricular fibrillation; VT, ventricular tachyarrhythmia

**Fig. 3:**
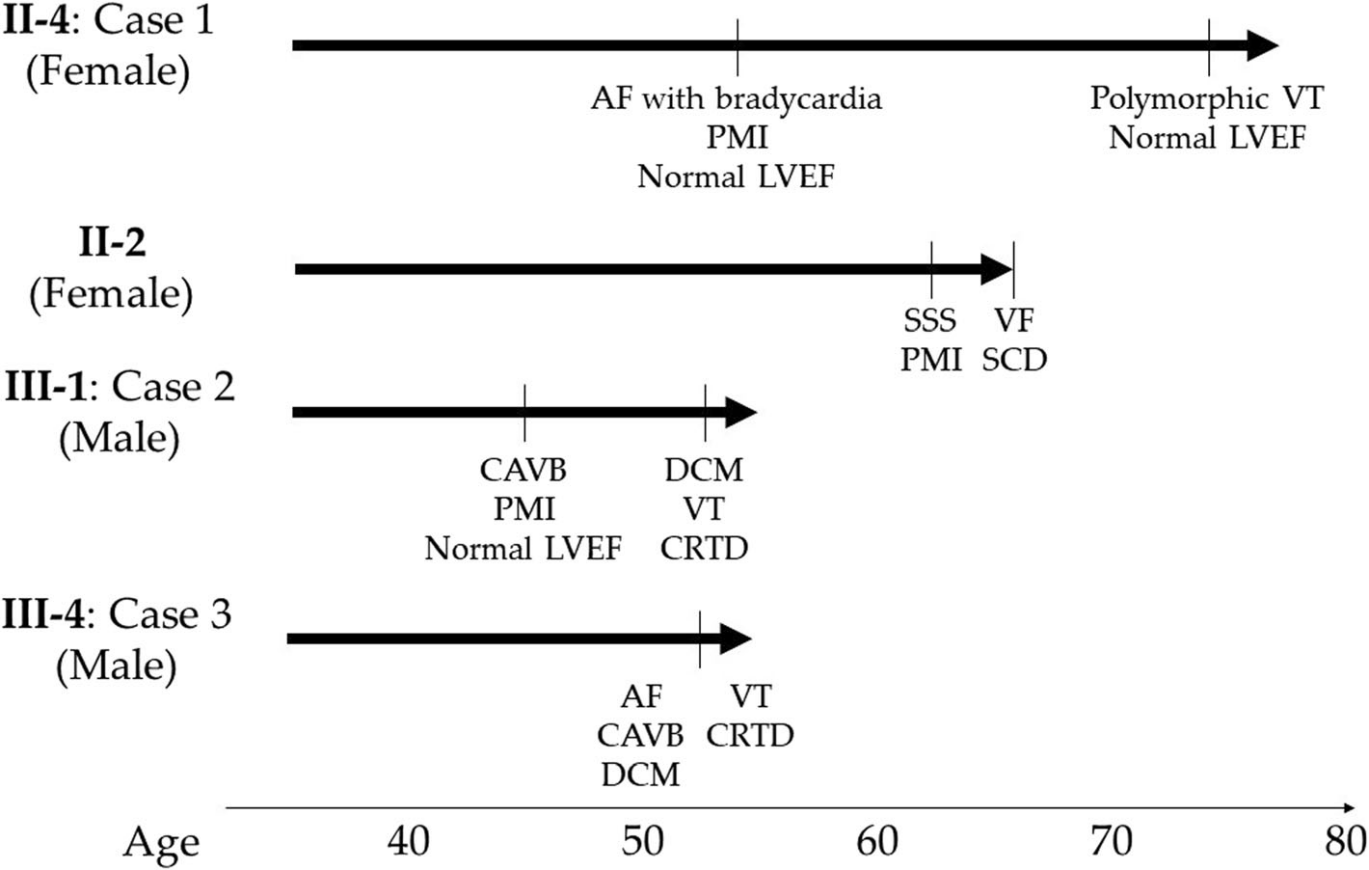
Clinical courses. The patient in Case 1 (female, **II-4,** proband) presented with AF with bradycardia in her fifties, and polymorphic VT in her seventies. The elder sister (**II-2**) of the patient in Case 1, who did not undergo genetic testing, presented with SSS with PMI in her sixties, and died suddenly due to VF. The patient in Case 2 (male, **III-1**) presented with CAVB in his forties, and DCM and VT in his fifties. The patient in Case 3 (male, **III-4**) presented with AF, CAVB, DCM, and VT in his fifties. AF, atrial fibrillation; CAVB, complete atrioventricular block; CRTD, cardiac resynchronization therapy defibrillator; DCM, dilated cardiomyopathy; LVEF, left ventricular ejection fraction; PMI, pacemaker implantation; SCD, sudden cardiac death; SSS, sick sinus syndrome; VF, ventricular fibrillation; VT, ventricular tachyarrhythmia

The patient’s family history suggested familial cardiac disease, including complete atrioventricular block, atrial fibrillation, VT, pacemaker implantation, SCD, DCM, and cardiac resynchronization therapy defibrillator (CRTD) implantation (Fig. 2a). Genetic testing was performed as described previously [15, 18], on the patient, her sons (**III-4**, details were described as Case 3, and **III-6**), and her nephew (**III-1**, details were described as Case 2). The details of the genetic testing are described in the Supplementary Materials. In brief, genomic DNA was extracted from blood leukocytes. Next-generation sequencing was performed for 56 genes associated with inherited primary arrhythmia syndromes, cardiomyopathy, and *LMNA*. Then, *LMNA* was detected in the patient’s family members. We developed polymerase chain reaction primers to amplify the protein-coding exons of *LMNA* for mutational screening. Genetic variant databases (NCBI NC_000001; NCBI NM_170707; NCBI NP_733821; NCBI NM_005572; NCBI NP_005563) were used for searches for the presence of the *LMNA* variants identified in the patients. An RNA splice site prediction tool (Berkeley Drosophila Genome Project: http://www.fruitfly.org) and in silico prediction tools of Polyphen2 (http://genetics.bwh.harvard.edu/pph2/), SIFT (http://sift.jcvi.org/) and Condel (http://bg.upf.edu/fannsdb/) were also used. We then discovered an *LMNA* nonsense mutation, c.475G > T, p.E159*, (the ClinVar accession number, SCV000996024) in the patient, her eldest son, and her nephew (Fig. 2b, c). Her second son did not have any *LMNA* mutations. The patient’s mother (**I-2**) had SCD in her fifties, and her older sister (**II-2,** clinical course was described in Fig. 3) was implanted with a pacemaker due to sick sinus syndrome in her sixties, 3 years after which she suffered ventricular fibrillation and died suddenly.

In Case 1 (**II-4**), implantation of a cardiovascular defibrillator (ICD) was recommended because of polymorphic VT, *LMNA* mutation, and family history of SCD. However, she refused the implantation and was discharged.

### Case 2

**Fig. 4:**
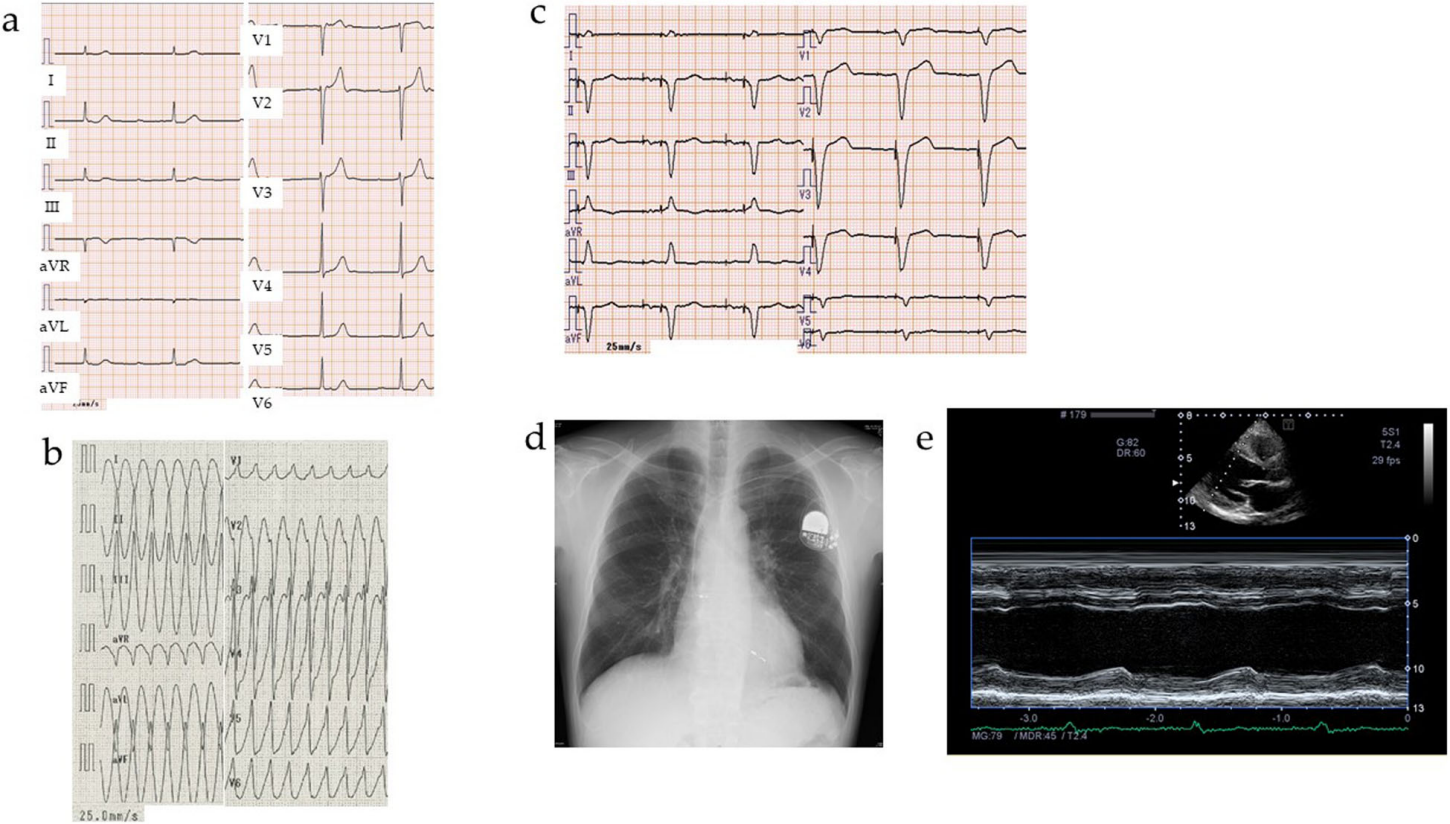
Images of Case 2 (**III-1**). **a** Case 2 developed complete atrioventricular block in his forties. **b** Five years after implantation of a pacemaker, he was admitted to hospital due to ventricular tachycardia. **c** On admission, electrocardiogram showed pacing rhythm, a heart rate of 60 ppm, and a QTc interval of 480 msec. **d** Chest X-ray revealed no congestion or pleural effusion, and a cardiothoracic ratio of 48.5% with a pacemaker. **e** Echocardiogram showed a reduced left ventricular ejection fraction of 33.2%, a large left ventricular diastolic diameter of 54.1 mm, and a left atrial diameter of 35.6 mm

A man (nephew of the patient in Case 1, **III-1**) in his forties developed complete atrioventricular block (Fig. 4a). Echocardiogram revealed normal LV function, and he was implanted with a pacemaker. Five years later he was admitted to hospital with VT (Fig. 4b). Echocardiogram revealed a reduced LV ejection fraction of 42.7%, and coronary angiography showed no significant stenosis of his coronary arteries. He was diagnosed as having DCM, and medical treatment with amiodarone, carvedilol, and imidapril was initiated. In the same year, he underwent genetic testing, and the same *LMNA* nonsense mutation seen in Case 1 was detected. He had an indication for CRTD implantation, and was referred to our hospital.

On admission, his blood pressure and heart rate were 118/72 mmHg and 60 ppm, respectively, and physical examination showed no sign of skeletal muscle disease. Laboratory findings revealed a B-type natriuretic peptide level of 59.5 pg/mL and a high troponin I level of 0.176 ng/mL (normal range: 0.000 to 0.056 ng/mL), as shown in Table 1. Electrocardiogram showed pacing rhythm, and chest X-ray showed no sign of congestion (Fig. 4c, d). Echocardiogram detected a reduced LV ejection fraction of 33.2% (Fig. 4e). Implantation of a CRTD, which was an upgrade from the patient’s pacemaker, was successfully performed. In the cardiopulmonary exercise test performed before discharge, his peak oxygen uptake was extremely low, at 10.3 mL/kg/min. After discharge, VT did not recur over a six-month follow-up period.

### Case 3

**Fig. 5:**
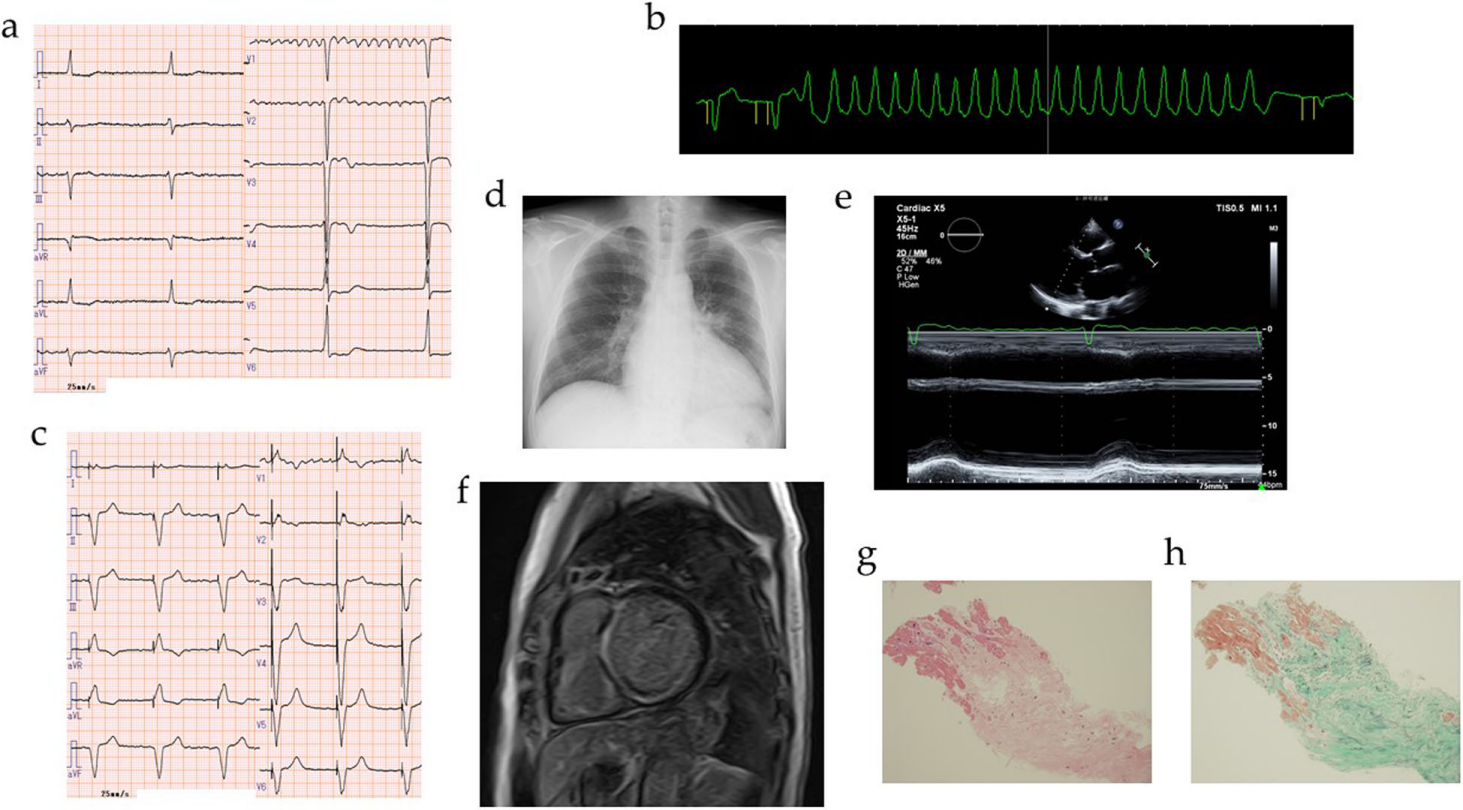
Images of Case 3 (**III-4**). **a** On admission, electrocardiogram showed atrial fibrillation with complete atrioventricular block and a heart rate of 40 bpm. **b** Monitor electrocardiogram revealed non-sustained ventricular tachycardia. **c** After the implantation of a cardiac resynchronization therapy defibrillator, electrocardiogram showed pacing rhythm, and a heart rate of 60 ppm. **d** Chest X-ray showed slight congestion with a cardiothoracic ratio of 60.4%. **e** Echocardiogram showed a reduced left ventricular ejection fraction of 33.1%, a large left ventricular diastolic diameter of 62.4 mm, and a large left atrial diameter of 45.9 mm. **f** Cardiovascular magnetic resonance showed late gadolinium enhancement in the left ventricle. **g**, **h** Left ventricular endomyocardial biopsy revealed interstitial fibrosis by Hematoxylin and eosin staining (g; magnification, × 50) and Elastica-Masson staining (h; magnification, × 50), but no significant findings of myocarditis, amyloidosis, or sarcoidosis

A man (eldest son of the patient in Case 1, **III-4**) in his fifties complaining of shortness of breath was admitted to a hospital due to heart failure with reduced LV ejection fraction. He was referred to our hospital for heart failure treatment. On admission, his blood pressure and heart rate were 122/68 mmHg and 40 bpm, respectively, and physical examination showed no sign of skeletal muscle disease. Laboratory findings revealed a high B-type natriuretic peptide level of 317.4 pg/mL and a high troponin I level of 0.215 ng/mL, as shown in Table 1. Electrocardiogram showed atrial fibrillation with complete atrioventricular block, and a heart rate of 40 bpm (Fig. 5a). Chest X-ray showed slight congestion with a cardiothoracic ratio of 60.4% (Fig. 5d). Echocardiogram detected a reduced LV ejection fraction of 33.1% (Fig. 5e). Treatment with enalapril, carvedilol, anticoagulant, and diuretics was initiated. After treatment for heart failure, cardiac catheterization was performed. Coronary angiography showed no significant stenosis of the patient’s coronary arteries. A right-sided catheter revealed a mean right atrial pressure of 8 mmHg, a mean pulmonary artery pressure of 18 mmHg, a mean pulmonary artery wedge pressure of 13 mmHg, and a cardiac index of 2.03 L/min/m^2^. Cardiovascular magnetic resonance showed late gadolinium enhancement in the left ventricle (Fig. 5f). Endomyocardial biopsy revealed interstitial fibrosis, but no significant findings of myocarditis, amyloidosis, or sarcoidosis (Fig. 5g, h). The patient was diagnosed as having DCM. Monitor electrocardiogram revealed repetitive non-sustained VT (Fig. 5b), and implantation of a CRTD was successfully performed. Electrocardiogram after CRTD implantation is described in Fig. 5c. He also underwent genetic testing, and the same *LMNA* nonsense mutation seen in Case 1 was detected.

The clinical courses of Case 1 (**II-4**, female), 2 (**III-1**, male), 3 (**III-4**, male), and the older sister of the patient in Case 1 (**II-2**) are shown in Fig. 3.

All parameters of the laboratory findings, electrocardiogram, chest X-ray, echocardiography, magnetic resonance imaging, cardiac pathology, and cardiac catheterization, were obtained from the hospital archive.

## Discussion and conclusions

The current study described five individuals, among one family, with conduction disorder, sick sinus syndrome, DCM, VT, and SCD, three of whom were available for testing, studied in detail, and shown to have the E159* *LMNA* mutation. Although the same mutation was briefly reported in a previous paper, the present report is the first to reveal detailed clinical presentations of family members, including those with the E159* *LMNA* mutation [15].

Truncation mutations like the E159* *LMNA* mutation are a risk factor for early onset of cardiac conduction disease, VT, and reduced LV ejection fraction, compared with missense mutations [13, 18]. However, clinical variability regarding severity, penetrance, age at onset, and sex difference has been observed among patients with the same *LMNA* mutations, even in identical twins [5, 19]. Several studies have demonstrated that patients with *LMNA* mutations have a higher incidence of lethal ventricular arrhythmias related to SCD in males than in females [11, 13–15]. However, unlike patients with other *LMNA* mutations, in the current study, SCD and malignant VT occurred in both males (**III-1** and **III-4**) and females (**I-2**, **II-2**, and **II-4**) among family members with the E159* *LMNA* mutation. Having a history of SCD and VT in females is unusual, compared to other families with *LMNA* mutations. A previous multicenter study showed that male patients with *LMNA* mutations had a higher prevalence of end-stage heart failure than female patients with the same mutations [14]. Regarding LV function among family members with the E159* *LMNA* mutation, our male patients (**III-1** and **III-4**) had worse LV function. The female proband (**II-4**) did not exhibit manifestations of reduced LV ejection fraction.

Polymorphic VT developed in our female patient (**II-4**). To our knowledge, there has yet to be a report on polymorphic VT in patients with the *LMNA* mutation, although VT is a common finding among such patients. Inflammation caused by influenza as well as low potassium levels are considered to affect the manifestation of polymorphic VT. The arrhythmic substrate of a patient with the *LMNA* mutation could also be associated with the occurrence of polymorphic VT.

Only one patient (**III-4**) underwent endomyocardial biopsy and cardiovascular magnetic resonance among the family members. Interstitial myocardial fibrosis detected by endomyocardial biopsy, and late gadolinium enhancement observed in this patient are consistent with other reported cases of *LMNA* mutations [20, 21]. Cardiac magnetic resonance imaging was considered to be useful for evaluating myocardial fibrosis in patients with the E159* *LMNA* mutation.

The LMNA has three domains; a short globular N-terminal head, a central rod and a long globular C-terminal tail [22]. LMNA interacts with a lot of large chromatin domains and is involved in genomic organization, recruitment of epigenetic regulators, and gene expression [23]. The E159* *LMNA* nonsense mutation removes more than half of the alpha-helical coiled-coil forming rod domain, as well as all of the C-terminal domain (Fig. 2c). Patients with these effects have both one normal *LMNA* gene and one mutated gene, and therefore any disease association would be due to haploinsufficiency [17]. *LMNA* mutations result in altered structure and altered interactions with chromatin and nuclear proteins. The *LMNA* mutation position predicts the involvement of several organ systems [24]. *LMNA* mutations localized upstream of the nuclear localization signal (exons 1 to 6) and tail have been reported to have more cardiac organ involvement compared with other systems [22, 24]. Such mutations were reported to have a more malignant cardiac phenotype compared with downstream mutations [22]. The E159* *LMNA* mutation is localized in exon 2 and upstream of the nuclear localization signal as shown in Fig. 2c. These findings suggest that patients with E159* nonsense mutation have cardiac problems.

*LMNA* mutation carriers can be asymptomatic [10, 25], and genetic screening for *LMNA* mutations in family members is clinically important to evaluate the risk of heart failure, arrhythmia, and SCD. Such screenings are recommended by the European Heart Rhythm Association [17, 26]. In family members similar to those in the current report, genetic screening should be considered even if the subject is asymptomatic.

ICD implantation is recommended at an early stage for DCM patients with *LMNA* mutations, compared to those with other causes [27]. CRTD implantation is considered to be a treatment for heart failure and prevention of SCD in DCM patients with *LMNA* mutations and reduced LV systolic function [18, 28]. Among the current cases, we performed CRTD implantation for our male patients (**III-1** and **III-4**), because these patients had LV systolic dysfunction and VT.

A limitation of this study was that the genetic testing was performed in only four members of the family. Additional genetic testing on unaffected family members would strengthen the finding that the E159* *LMNA* mutation is associated with cardiac problems. Further study is required to demonstrate the causality between the E159* *LMNA* mutation and the cardiac problems.

In conclusion, clinical manifestations of various atrial and ventricular arrhythmias and heart failure with reduced left ventricular ejection fraction age-dependently occurred in family members carried with the E159* *LMNA* mutation. It appears highly likely that the E159* *LMNA* mutation is related to various cardiac problems, as characterized by the family of the current report.

## Supplementary information

**Additional file 1: **Supplementary Methods. **Table S1**. Genes for target screening.

## Data Availability

The data that support the findings in this study are available from Tetsuro Yokokawa upon reasonable request and permission of Fukushima Medical University.

## Abbreviations

CRTD: Cardiac resynchronization therapy defibrillator
DCM: Dilated cardiomyopathy
ICD: Implantable cardiovascular defibrillator
*LMNA*: Lamin A/C
LV: Left ventricular
SCD: Sudden cardiac death
VT: Ventricular tachyarrhythmia
