# Supplementary material for: Case reports of a c.475G>T, p.E159* lamin A/C mutation with a family history of conduction disorder, dilated cardiomyopathy and sudden cardiac death
Source: BMC Cardiovasc Disord. 2019 Dec 17;19:298. doi: 10.1186/s12872-019-01282-6 (PMC6918565; doi:10.1186/s12872-019-01282-6)
Supplement: Supplementary file 1 — Additional file 1: Supplementary Methods. Table S1. Genes for target screening. [file 12872_2019_1282_MOESM1_ESM.docx]

**Supplementary Materials**

**Yokokawa T, et al.**

**Case reports of a c.475G>T, p.E159* lamin A/C mutation with a family history of conduction disorder, dilated cardiomyopathy and sudden cardiac death**

**Supplementary Methods**

**Genetic analysis**

Genomic DNA was extracted from peripheral blood leukocytes using a DNA isolation kit for Mammalian Blood (Roche Diagnostics, Basel, Switzerland).

All the patients included in this study were screened by next-generation sequencing MiSeq (Illumina, CA, USA). A TruSight Cardio Sequencing Kit (Illumina) or a customized Haloplex HS panel kit (Agilent Technology, CA, USA) was used for choosing 56 genes associated with inherited primary arrhythmia syndromes, including long-QT-syndrome-related genes, Brugada-related genes, catecholaminergic polymorphic ventricular tachycardia, arrhythmogenic right ventricular cardiomyopathy, some hypertrophic cardiomyopathy/dilated cardiomyopathy, and *LMNA* (Supplementary Table 1). The obtained data were analyzed using SureCall software (Agilent Technology, CA, USA).

Detected variants were confirmed by the Sanger method.

Standard polymerase chain reaction (PCR) primers were derived from intronic sequences to amplify the 12 protein-coding exons of *LMNA*. Mutational screenings of PCR amplicons were performed by direct sequencing on an ABI PRISM 3130x Genetic Analyzer (Thermo Fisher Scientific, Waltham, Massachusetts) by using BigDye Terminator chemistry (v1.1 or 3.1) according to the standard protocols. The reference sequences used in this study were as follows: *LMNA* gene: NCBI NC_000001; *LMNA* messenger ribonucleic acid: NCBI NM_170707; lamin A protein: NCBI NP_733821; *LMNC* messenger ribonucleic acid: NCBI NM_005572; lamin C protein: NCBI NP_005563.

**Bioinformatic analysis**

Mutations present in dbSNP build 146 or published in previous literature were identified. All non-matching variants were filtered using a minor allele frequency threshold < 0.3% based on the Human Genetic Variation Database (http://www.genome.med.kyoto-u.ac.jp/SnpDB/), which includes the Japanese population, Exome Aggregation Consortium (http://exac.broadinstitute.org), and ClinVar (http://www.ncbi.nlm.nih.gov/clinvar/). All single base substitutions without changes in the coding amino acid were screened by a splice site prediction tool (Berkeley Drosophila Genome Project: http://www.fruitfly.org), and the possibility of aberrant splicing was estimated. The candidate variant was considered a pathogenic mutation if it generated a stop codon, a frameshift of the open reading frame, or an aberrant splice site. The pathogenicity of novel missense variants was predicted by in silico analysis using Polyphen2 (http://genetics.bwh.harvard.edu/pph2/), SIFT (http://sift.jcvi.org/), and Condel (http://bg.upf.edu/fannsdb/). A novel missense variant was considered pathogenic if classified as ‘probably damaging’ by Polyphen2, ‘damaging’ by SIFT, or predicted to be ‘deleterious’ by Condel.

**Table S1. Genes for target screening**

| *KCNQ1* | *KCNJ8* | *MYH7* | *LDB3* |
| --- | --- | --- | --- |
| *KCNH2* | *CACNA2D1* | *TNNT2* | *PLN* |
| *SCN5A* | *KCND3* | *TPM1* | *CRYAB* |
| *Ankyrin-B* | *ABCC9* | *MYBPC3* | *FHL2* |
| *KCNE1* | *TRPM4* | *MYL3* | *MYPN* |
| *KCNE2* | *GJA5* | *MYL2* | *NEXN* |
| *KCNJ2* | *RYR2* | *TNNI3* | *BAG3* |
| *CACNA1C* | *CASQ2* | *ACTC* | *DMD* |
| *CAV3* | *TRDN* | *TTN* | *EMD* |
| *SCN4B* | *CALM1* | *MYH6* | *TAZ* |
| *AKAP-9* | *LMNA* | *CSRP3* | *FKTN* |
| *SNTA1* | *DSP* | *TCAP* | *TGFB3* |
| *KCNJ5* | *PKP2* | *VCL* | *TMEM43* |
| *CACNB2* | *DSG2* | *JPH2* | *DTNA* |
| *GPD1L* | *DSC2* | *MYOZ2* | *PRKAG2* |
| *SCN1B* | *JUP* | *ANKRD1* | *GAA* |
| *KCNE3* | *HCN4* | *DES* | *GLA* |
| *SCN3B* | *TBX5* | *ACTN2* | *LAMP2* |
